# Editorial: From bench to bedside: inflammation in neurovascular disorders and stroke

**DOI:** 10.3389/fnhum.2025.1658045

**Published:** 2025-07-17

**Authors:** Mohd Farooq Shaikh, Rony Abdi Syahputra, Mohd Kaisan Mahadi

**Affiliations:** ^1^School of Dentistry and Medical Sciences, Charles Sturt University, Orange, NSW, Australia; ^2^Department of Pharmacology, Faculty of Pharmacy, Universitas Sumatera Utara, Medan, Indonesia; ^3^Centre for Drug and Herbal Development, Faculty of Pharmacy, Universiti Kebangsaan Malaysia, Kuala Lumpur, Malaysia

**Keywords:** ischemic stroke, ischemic reperfusion brain injury, hemorrhagic stroke, inflammation, neurovascular disorder

This Research Topic aims to deepen the understanding of inflammatory processes underlying neurovascular diseases, such as stroke, by emphasizing the identification of specific inflammatory biomarkers, elucidation of molecular pathways in cerebral ischemia-reperfusion injury, and exploration of novel treatment strategies.

Ischemia-reperfusion (I/R) injury refers to the paradoxical worsening of cellular dysfunction that occurs following the restoration of blood flow after an ischemic stroke. A study by Deng et al. demonstrates a strong association between the inflammatory marker neutrophil-albumin ratio and poor clinical outcomes in stroke patients post-thrombolysis, suggesting its predictive capability in determining I/R injury. A notable manifestation of I/R injury is the development of hemorrhagic transformation (HT) and symptomatic intracranial hemorrhage (sICH). Bao et al. investigated the role of peripheral immune inflammation in HT and sICH following endovascular thrombectomy in 81 acute ischemic stroke patients. Their study identified interleukin-6 (IL-6) and the neutrophil-to-albumin ratio as promising biomarkers for predicting complications after endovascular thrombectomy, thereby aiding in prognosis and clinical management. Similarly, Qian et al. conducted a retrospective analysis of 323 patients with large artery occlusion who were treated with mechanical thrombectomy. They found that an elevated systemic immune-inflammation index on admission was a significant predictor of HT, malignant brain oedema, poor 90-day functional outcomes, and mortality. These findings highlight the prognostic value of the neutrophil-albumin ratio and the systemic immune inflammation index, suggesting that targeting inflammation may improve outcomes following reperfusion therapy.

Inflammatory responses determine the pathogenesis and severity of ischemic stroke injury. In experimental models, focal cerebral ischemia triggers a progressive recruitment and activation of inflammatory cells, including neutrophils, T lymphocytes, and monocytes/macrophages, to facilitate tissue repair and regeneration (Yoshimura and Ito, 2020; Alsbrook et al., 2023; Hasan et al., 2024). Li et al.  employed bioinformatics to identify 33 nucleotide metabolism-related genes associated with immune and inflammatory pathways in ischemic stroke, as retrieved from the GEO database. Among these, *CFL1, HMCES*, and *GIMAP1* emerged as key immune-related genes with strong diagnostic potential and associations with immune cell infiltration. Subsequent drug-target prediction, single-cell RNA sequencing, molecular docking, and *in vivo* validation reinforced these findings. Exosomes which are small vesicles released by cells to mediate intercellular signaling, have been shown to influence the inflammatory response after stroke. Zhao et al. integrated multiomics data from five ischemic stroke datasets with exosome-related genes, applying machine learning and network analyses to identify 13 features and 10 hub genes. Notably, LGALS3, CD36, TLR2, ICAM1, and CD14 were upregulated in mouse stroke models, implicating inflammatory, chemokine, and JAK–STAT signaling pathways. Immune infiltration analysis revealed altered immune cell populations, with CD14 and LGALS3 linked to monocytes/macrophages and neutrophil activity. Single-cell analysis highlighted microglial dynamics, positioning exosomal genes as promising therapeutic targets in ischemic stroke.

In hypertensive patients, immune-inflammatory responses significantly influence stroke prognosis. A cross-sectional study by Tang et al., which included 9,699 adults from the National Health and Nutrition Examination Survey (2001–2016), found that the systemic immune response index (SIRI) is a strong predictor of stroke risk, especially among Chinese males over 20 years of age. This finding highlights SIRI's potential as a target for therapeutic intervention and stroke prevention. Wang et al. developed a nomogram combining SIRI and clinical factors to predict short-term outcomes in very elderly patients with hypertensive intracerebral hemorrhage (HICH). Analysis of 324 patients identified the Glasgow Coma Scale score, hematoma expansion, chronic obstructive pulmonary disease, and systemic immune response index as independent predictors of poor prognosis. The nomogram demonstrated excellent predictive accuracy, supporting its clinical utility. Similarly, Liu et al. reported a positive linear correlation between neutrophil-lymphocyte ratio and perihematomal oedema in hypertensive cerebral hemorrhage, indicating this inflammatory marker as a prognostic indicator.

Targeting inflammation offers therapeutic promise in stroke management. Nevertheless, suppressing the inflammatory responses has been suggested to reduce the size of the infarct and enhance neurological recovery in ischemic stroke injury (Mizuma and Yenari, 2017). Corbali and Nahab indicated that colchicine, an NLRP3 inflammasome inhibitor, could reduce stroke risk in cardiovascular patients under the age of 65. However, stroke-specific trials (CONVINCE, CHANCE-3) yielded limited or no significant benefit. Low-dose colchicine (0.5 mg daily) is generally safe but may cause myopathy, particularly with statins. Current guidelines cautiously endorse colchicine for atherosclerotic stroke prevention, pending further validation. Nevertheless, it is also essential to consider that the latest Cochrane stroke group reported that randomized trials on anti-inflammatory therapy did not produce any benefits against recurrent stroke (Coveney et al., 2020). A preclinical study by Cao W. et al. highlights cynaroside (Cyn) as a potential neuroprotective agent. Cyn treatment improved neurological outcomes, reduced infarct size, oedema, and microglial activation in ischemia/reperfusion models by inhibiting arachidonate 15-lipoxygenase (Alox15), a key enzyme in inflammation and ferroptosis. This inhibition decreased pro-inflammatory cytokines (NLRP3, IL-1β, and IL-18) and ferroptosis-related proteins (Tfrc, COX2, and Acsl4), suggesting that Cyn mitigates ischemic injury via anti-inflammatory and anti-ferroptotic mechanisms. Further research is warranted to confirm these therapeutic effects and clinical applicability.

Integrating multiple modalities is necessary in managing stroke associated injuries. Cao Y. et al. develop and validate a machine learning-based model to predict stroke-associated pneumonia (SAP) in older adults with hemorrhagic stroke. Analysis performed on clinical data from 788 patients across multiple centers, comparing four algorithms: XGBoost, Logistic Regression, Support Vector Machine (SVM), and Naive Bayes. Key risk factors identified included advanced age, smoking history, lower Glasgow Coma Scale and Braden scores, and use of nasogastric tubes. The model was visualized via a nomogram to enable rapid bedside risk assessment, facilitating early identification and targeted prevention to reduce SAP incidence and improve outcomes. In another study, Liao et al. evaluated CTA angiographic point sign-guided stereotactic surgery in 143 patients with moderate basal ganglia hematoma. Compared to CT-guided surgery, the CTA-guided approach significantly reduced secondary hematoma expansion (0 vs. 18.75%), lowered mortality (2.53 vs. 12.5%), decreased lung infections, and improved 6-month functional outcomes, demonstrating enhanced safety and efficacy. He et al. examined 158 stroke neurosyphilis patients treated with combined hyperbaric oxygen (HBO) and transcranial ultrasound neuromuscular stimulation (TUS-NMES) showed significant improvements in motor, cognitive, and daily living functions. Decreased inflammatory marker CXCL13 levels correlated with recovery, and a biomarker panel of CXCL13, WBC, and Hs-CRP effectively predicted rehabilitation outcomes, supporting their clinical use in managing neurosyphilis stroke recovery.

In conclusion, understanding and targeting inflammatory pathways via multiple modalities offers a promising therapeutic avenue for mitigating brain injury associated with stroke. Emerging evidence from therapies like colchicine and cynaroside highlights their potential to modulate inflammation; however, further rigorous investigation is crucial to establish their definitive benefits and safety for patient application. Moving forward, the synergistic integration of insights from basic laboratory research and clinical studies will be essential for developing personalized treatments that significantly enhance outcomes for individuals affected by stroke.
